# Clinical Appraisal of “Micronized Vaginal Progesterone Dose and Serum Progesterone Thresholds Determine Reproductive Outcomes in Frozen–Thawed Embryo Transfer With Hormone Replacement Therapy”

**DOI:** 10.1002/rmb2.70088

**Published:** 2026-08-19

**Authors:** I‐Hsuan Wu, Juan‐Enrique Schwarze, Christoph Helwig, Thomas D'Hooghe, Ilka Schellschmidt

**Affiliations:** ^1^ Merck Healthcare KGaA Darmstadt Germany; ^2^ Global Medical Affairs Fertility, Research and Development, Merck Healthcare KGaA Darmstadt Germany; ^3^ Department of Development and Regeneration, Laboratory of Endometrium, Endometriosis & Reproductive Medicine KU Leuven Leuven Belgium; ^4^ Department of Obstetrics, Gynecology, and Reproductive Sciences Yale University Medical School New Haven Connecticut USA

## Abstract

This paper critically evaluates Sekiguchi et al.'s study of vaginal progesterone dose, serum progesterone thresholds, and reproductive outcomes in hormone replacement therapy frozen embryo transfer (HRT‐FET) cycles. The study conflates descriptive, predictive, and causal aims without a clearly prespecified primary question, limiting interpretability. Major concerns include non‐random treatment allocation, potential residual confounding, absence of a causal framework for covariate selection, unaddressed temporal confounding related to Japan's 2022 insurance reform, and exclusion of an available progesterone formulation. Additionally, formulation specific pharmacokinetics, single‐time‐point progesterone measurement, and internally derived thresholding limit comparability, predictive validity, and clinical applicability.

We read with interest the article by Sekiguchi et al. on micronized vaginal progesterone dose, serum progesterone thresholds, and reproductive outcomes in HRT‐FET cycles [1]. The authors concluded that higher vaginal progesterone doses and higher serum progesterone concentrations on the day of embryo transfer were associated with improved reproductive outcomes, including live birth.

While the topic is clinically relevant, several aspects of the study design and analysis warrant careful consideration. In our view, these limitations restrict the extent to which causal or practice‐changing conclusions can be drawn and may also affect generalizability.

A major concern is the absence of a clearly defined research question. The study combines descriptive, predictive, and causal objectives, although each requires different methodological assumptions and analytical approaches. Without a prespecified primary objective, it is difficult to determine whether the analyses should be interpreted as descriptive comparisons, prediction modeling, or causal inference. As a result, the conclusions exceed what can reasonably be supported by retrospective observational data.

If the authors intend to infer that progesterone dose or serum progesterone concentrations influence reproductive outcomes, the assumptions required for causal inference, particularly exchangeability, positivity, and consistency, should be explicitly addressed. However, these assumptions are not sufficiently discussed.

The progesterone formulation was selected by the patient after receiving information on the available products. Women selecting or being guided toward one formulation may have differed systematically from those receiving another formulation in baseline or clinical characteristics also related to reproductive outcomes. This threatens exchangeability and limits the extent to which differences in implantation, clinical pregnancy, ongoing pregnancy, or live birth rates can be attributed to the progesterone regimen itself.

Similarly, the covariates included in the multivariable regression model do not appear to have been selected according to a prespecified causal framework. In causal analyses, adjustment variables should ideally be chosen based on subject matter knowledge and represented through a directed acyclic graph [2]. Without such a framework, it remains unclear whether important confounders were omitted or whether inappropriate variables were adjusted for. This substantially limits causal interpretation of the observed associations [3].

The study period extended from 2017 to 2022, during which assisted reproductive technology (ART) treatment in Japan became covered by national health insurance in April 2022. This policy change may have altered patient access and preferences, treatment patterns, and clinical practice [4]. Calendar year, or at least treatment period before versus after April 2022, should therefore be considered as an additional covariate or sensitivity analysis, particularly because the article shows variation in progesterone formulation use over time.

Another point requiring clarification is the selection of progesterone formulations included in the analysis. Although four vaginal progesterone products are currently available in Japan, only three regimens, P(90), P(300), and P(800), were analysed [5]. The rationale for excluding the P(600) formulation is not provided. This omission may have influenced both the observed dose–serum progesterone relationship and the apparent association between progesterone regimen and reproductive outcomes.

Direct comparisons based solely on nominal daily progesterone dose should also be interpreted cautiously. The analyzed formulations differ not only in dose but also in pharmaceutical form, namely gel, tablet, and capsule, each with distinct pharmacokinetic properties. Therefore, the observed differences between P(90), P(300), and P(800) cannot necessarily be attributed to dose alone and may instead reflect formulation‐specific pharmacology, administration frequency, timing of blood sampling, or patient adherence.

The predictive analyses also warrant careful interpretation. In prediction research, the key issue is not whether a variable is statistically associated with an outcome, but whether it improves prediction beyond an appropriate clinical model. However, the authors relied mainly on ROC analysis of serum P4 as a single marker and derived an “optimal” cut‐off of 13.1 ng/mL using the Youden index. Although this approach identifies the threshold maximizing sensitivity and specificity within the analyzed dataset, it does not establish whether serum P4 has clinically meaningful predictive value. This is particularly important because the reported area under the curve (AUC) was only 0.54 (95% CI 0.51–0.58), indicating discriminative performance only marginally better than chance. Consequently, the clinical relevance of the proposed threshold remains questionable.

This concern is compounded by the fact that the cut‐off was both derived and evaluated in the same dataset. Dichotomizing the cohort according to this internally derived threshold and subsequently comparing outcomes above and below 13.1 ng/mL led to overfitting and optimistic estimates of association. Without external validation, or at least internal validation using appropriate resampling methods, the robustness and generalizability of this threshold remain unclear.

Although the authors attempted to standardize the timing of serum progesterone measurement on the day of embryo transfer, serum P4 was assessed at a single time‐point only. Samples were obtained 6 h after administration for OneCrinone and Luteum, and immediately after administration for Lutinus. Given the known intraday and interindividual variability in serum progesterone concentrations, a single measurement may capture different points along the concentration–time profile for each formulation.

This issue is particularly relevant because the analyzed products differ in pharmaceutical form and pharmacokinetic profile. As a result, a single serum P4 value may not be directly comparable across formulations, even when sampling follows a predefined schedule. In addition, progesterone was self‐administered at home, making the actual timing of administration difficult to verify in routine clinical practice. Any deviation from the assumed dosing time could have influenced serum P4 levels and the classification of patients above or below the proposed threshold.

In conclusion, Sekiguchi et al. address an important question regarding individualized luteal phase support in HRT‐FET cycles. However, the retrospective design, non‐random treatment allocation, formulation‐specific pharmacokinetic differences, and limitations in the predictive and causal analyses warrant cautious interpretation. Overall, the study should primarily be viewed as exploratory and hypothesis generating rather than as evidence supporting specific serum progesterone thresholds or dose‐based clinical strategies. Future prospective studies using clearly defined causal or predictive frameworks, standardized progesterone monitoring, and independent validation cohorts are needed to determine the true clinical utility of serum progesterone guided luteal phase support.

## Funding

Financial support was provided by Merck KGaA, Darmstadt, Germany.

## Ethics Statement

The authors have nothing to report.

## Consent

The authors have nothing to report.

## Conflicts of Interest

I‐Hsuan Wu, Juan‐Enrique Schwarze, Ilka Schellschmidt, Christoph Helwig, and Thomas D'Hooghe are employees of Merck Healthcare KGaA, Darmstadt, Germany. Christoph Helwig owns shares of Merck KGaA.

## Data Availability

Data sharing is not applicable to this article as no datasets were generated or analyzed during the current study.
